# Cetuximab-based therapy in elderly comorbid patients with metastatic colorectal cancer

**DOI:** 10.1038/bjc.2011.554

**Published:** 2012-01-03

**Authors:** C F Jehn, L Böning, H Kröning, K Possinger, D Lüftner

**Affiliations:** 1 Medizinische Klinik und Poliklinik m. S. Onkologie & Hämatologie; Charité Campus Mitte, Universitätsmedizin Berlin, Charitéplatz 1, 10117 Berlin, Germany; 2 Onkologische Praxis Elisenhof, München, Prielmayerstraße 1, 80335 München, Germany; 3 Gemeinschaftspraxis für Hämatologie und Onkologie, Hasselbachplatz 2, 39104 Magdeburg, Germany

**Keywords:** cetuximab, chemotherapy, metastatic colorectal cancer, elderly patients

## Abstract

**Background:**

Clinical trials under-represent patients (pts) >65 years. Non-interventional studies (NISs) help to evaluate therapies in daily practice. This NIS evaluates efficacy and safety of cetuximab in combination with chemotherapy in metastatic colorectal cancer (mCRC) pts aged >65 years *vs* ⩽65 years.

**Methods:**

A total of 657 pts were recruited into the NIS and analysed applying descriptive statistics and *χ*^2^ or Fisher's exact test.

**Results:**

A total of 309 and 305 pts aged ⩽65 and >65 years, respectively, were documented; 80% showing a reduced ECOG status of 1–2 and 95% having received at least one palliative treatment. Cetuximab was combined with irinotecan according to approval status. Grade III/IV toxicities occurred in 20% of pts without any difference between age groups although the older pts had significantly more pre-existing comorbidities (*P*=0.001). A total of 64.2% of the pts developed skin rash, which was strongly related to response (*P*<0.0002) without any difference between age groups (*P*=0.34). The objective response rates were 37.9% for ages 18–65 years *vs* 35.4% for >65 years. Progression-free survival (PFS) did not differ between pts 18–65 years old (6.5 months) in comparison with pts >65 years (7.0 months). In a multivariate analysis only ECOG status had a negative impact on PFS (HR: 0,675; 95% Cl, 0.53–0.87; *P*=0.0019).

**Conclusion:**

This NIS reports one of the largest mCRC collectives >65 years and reduced performance status. Cetuximab has a similar efficacy and safety profile for pts aged ⩽65 and >65 years.

Even though significant advances in the treatment of colorectal cancer (CRC) were made in the last decade with combination chemotherapy, including fluorouracil, irinotecan and oxaliplatin, CRC remains the second most leading cause of cancer-related death in the United States and western Europe (Ries *et al*, 2010). The development of new therapeutic agents, which target specific molecular events in tumour cells, provide new opportunities to improve treatment of this type of cancer (Imai and Takaoka, 2006). One such agent is the epidermal growth factor receptor (EGFR)-targeted IgG monoclonal antibody cetuximab (*Erbitux*; Merck, Darmstadt, Germany). Cetuximab interacts with the extracellular domain of EGFR, thereby partially occluding the ligand-binding region, and sterically preventing the receptor from modulating conformation required for dimerisation and active signalling. Furthermore, it is suspected that an antitumour, antibody-dependent, cell-mediated cytotoxic reaction is triggered (Li *et al*, 2005; Zhang *et al*, 2007). The efficacy of cetuximab was shown in various studies like the multinational EPIC, the BOND and the MABEL study in the irinotecan-refractory setting of pretreated metastatic colorectal cancer (mCRC). In the EPIC trial involving 1298 patients (pts) after oxaliplatin-based chemotherapy, cetuximab added to irinotecan *vs* irinotecan alone significantly improved progression-free survival (PFS) (median, 4.0 *vs* 2.6 months) and quality of life analysis (*P*=0.047) (Sobrero *et al*, 2008). In the Bond trial (329 pts), the combination of cetuximab plus irinotecan showed an improved response rate (RR) of 23% and a prolonged time to progression (TTP) of 4.1 months compared with cetuximab monotherapy (RR 10.8% and TTP 1.5 months) in irinotecan-refractory pts (Cunningham *et al*, 2004). Consistent with these findings, the MABEL study (1147 pts) demonstrated, in a wider standard community practice setting, similar efficacy and safety with a RR of 20% and a median overall survival of 9.2 months in pts whose mCRC had progressed on irinotecan-based chemotherapy when treated with Cetuximab in combination with a irinotecan-based chemotherapy (Wilke *et al*, 2008). Both studies included mainly pts with a good performance status (ECOG 0–1) and a median age in the Bond trial of 59 years and of 62 years in the MABEL study. In our experience, these pts do not represent the usual population at such advanced stage and line of pretreatment of mCRC in standard general practice. This German non-interventional study (NIS) evaluated the efficacy and safety of cetuximab in combination with chemotherapy in pretreated mCRC in pts with reduced performance status and aged >65 years.

## Materials and methods

This was an uncontrolled multicentre study, which recruited mCRC pts in an outpatient setting at 87 study sites throughout Germany between April 2005 and November 2007. Informed consent was obtained from all subjects and/or their guardians. The data of 657 pts with mCRC were collected in an electronic documentation system (E-CRF). At the end of data collection, 614 complete data sets were available for analysis of efficacy and toxicity.

To be included, pts (>18 years of age) had to have histologically or cytologically confirmed EGFR-expressing mCRC. The significance of the k-RAS status was not established at the time of study accrual and hence no prerequisite for inclusion. Exclusion criteria were a history of hypersensitivity to cetuximab (grades III and IV), pregnancy and lactation. All other considerations were left to the discretion of the treating physician to maintain conditions typical for a wider standard community practice setting. Treatment consisted of cetuximab (400 mg m^−2^ week 1, followed by 250 mg m^−2^ weekly) either in combination with chemotherapy or alone as monotherapy. Three irinotecan regimes were applied: 350 mg m^−2^ every 3 weeks, 180 mg m^−2^ every 2 weeks and 80–125 mg m^−2^ weekly for 4 weeks followed by 2 weeks of rest, according to the approval status at that time. In addition to irinotecan, other chemotherapy agents like 5-fluorouracil (5-FU)/folinic acid, oxaliplatin, capecitabine, gemcitabine and mitomycin C were combined with cetuximab in various treatment lines.

Radiologic imaging was conducted at baseline and according to general practice every 8 weeks or when disease progression was suspected. Efficacy was assessed according to the WHO criteria (complete remission (CR), partial remission (PR), stable disease and progressive disease (PD)), including RR (CR and PR) and PFS. Complete response was defined as disappearance of all tumour manifestations with confirmation 4 weeks afterwards and PR was defined as >50% regression of measurable lesions. Progression-free survival was defined as the time in months between first cetuximab infusion and disease progression or death. Toxicity was graded according to the National Cancer Institute Common Toxicity Criteria (Trotti *et al*, 2003). In addition to the ECOG performance status, pre-exisiting comorbid conditions were recorded and a score according to the Charlson Comorbidity Index (CCI) was formed (Charlson *et al*, 1987). A 12-month observation period was set for this study.

We analysed both patient groups applying descriptive statistics. All results were considered significant at *P*<0.05 (two-tailed). For correlations of metric variables, Spearman rank order (rho), and for correlations of nominal variables, the *χ*^2^-test was used. To analyse the association between categorical variables and age groups (18–65 years and >65 years), the *χ*^2^-test, or for small sample sizes, the Fisher's Exact test were applied. The Wilcoxon–Mann–Whitney test for independent samples was used to analyse for differences between the age groups (18–65 *vs* >65 years). All results of the various statistical tests are of explorative nature. A multivariate analysis was performed to assess the influence of patient characteristics (CCI, age, age at diagnosis, gender, ECOG and location of primary tumour) on PFS.

## Results

A total of 614 pts were included into the analysis (Table 1). The median age of all pts was 65 years (range 23–89). Patients were divided into two age groups: the age group 18–65 included 309 pts (50.3%) with a median age of 59 years (range 23–65). The age group 66 years and older included 305 pts (49.7%) with a median age of 71 years (range 66–89). There was an equal distribution between men and women in both age groups (*P*=0.65, *χ*^2^-test). The median age at diagnosis with CRC was 62 years. In 60%, the primary tumour was located in the colon, whereas in 40%, the primary site was the rectum. In all, 78% of the pts had an ECOG performance status of 1 or 2, whereas 20% had an ECOG performance status of only 2 or 3. There was no difference in ECOG performance status between the two age groups (*P*=0.56, *χ*^2^-test).

Cardiovascular disease was the most frequent comorbidity. In all, 28.8% of the pts were affected, with the majority of pts in the older age group >65 years (*P*=0.0001, *χ*^2^-test), followed by diabetes (11.1%) and pulmonary disease (7.3%). The latter two pre-existing medical disorders showed no differences in frequency between the age groups. The older age group >65 years showed more comorbidities with a higher CCI, compared with the younger patient group (*P*=0.002, *χ*^2^-test) (Table 1). A multivariate analysis was performed to evaluate the influence of patient characteristics like gender, ECOG status, age, primary location of CRC and CCI on PFS. Only ECOG status had a significant negative influence on PFS (HR: 0,675; 95% Cl, 0.53–0.87; *P*=0.0019).

Regarding previous therapy, about 45% of the pts in both groups had received an adjuvant chemotherapy, while 95% of the pts had received at least one chemotherapy for metastatic disease (Table 2). In all, 39% of the pts had received two previous chemotherapies before cetuximab initiation. Close to one-fifth of the pts had received either one (22.5%) or even three (21.5%) previous chemotherapies. Compared with the older pts group (>65 years), more pts in the younger patient group (18–65 years) had received only one previous chemotherapy (*P*=0.025, *χ*^2^-test).

A total of 91.2% of the pts on study received chemotherapy in combination with cetuximab, whereas 8.8% pts were treated with cetuximab alone. Cetuximab was mainly combined with irinotecan (82%) in various doses according to the approval status at that time, in 25% the combination regimen contained 5-FU and in 4.6% oxaliplatin. Most pts (60%) received irinotecan on a weekly basis at a dose of 80 mg m^−2^, 15% of pts in a weekly dose (4 weeks, then 2 weeks of rest) of 125 mg m^−2^ and only 25% received doses at other than weekly intervals. There was no difference in terms of dosing or application frequency between the two age groups (*P*=0.51 and *P*=0.08, respectively, *χ*^2^-test). However, there was a trend in favour of a 2-weekly application in the younger patient group. Patients were treated for a median of 4 months with a median of 15 infusions of cetuximab. In 106 cases, a dose modification of cetuximab was implemented, in 30% of these cases the reason being skin toxicity. The major reason for treatment interruption was by request of the patient (23.6%). In 92% of the cases, therapy was terminated before the end of the 12-month observational period, tumour progression being the most frequent reason. One-third of the pts died during the study period.

**Figure 1:**
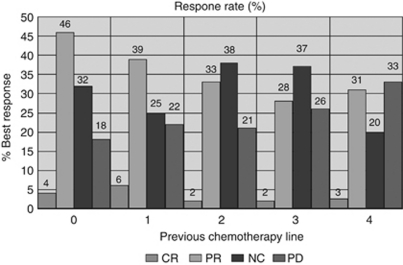
Best response in percentage of pts according to their previous line of chemotherapy (CR, PR, no change (NC) and PD).

**Figure 2:**
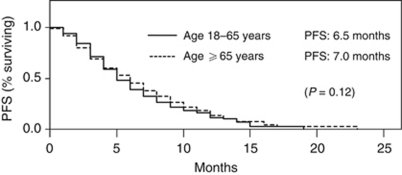
Progression-free survival of pts in age groups 18–65 years *vs* ⩾65 years clearly showing no difference between both patient subsets.

A total of 37% of all pts achieved an objective response to cetuximab-based treatment. Overall response rates (ORR) were highest for pts without or with a maximum of only one previous chemotherapeutic treatment (Figure 1). With regard to previous chemotherapy line and overall response, there were no differences between the two age groups: 38% for ages 18 ⩽65 years *vs* 36% for ages >65 years, (*P*=0.89, *χ*^2^-test). Cetuximab-based chemotherapy showed no difference in effectiveness between the pts who had previously received either oxaliplatin or irinotecan in a previous chemotherapy line (ORR: 36.2% *vs* 48.3%, respectively; *P*=0.19, *χ*^2^-test). Patients who achieved a CR or PR as best response showed in 90.8% of the cases any grade of skin reaction, compared with only 76.2% who did not respond (sum of the pts with stable disease or progressive disease) (*P*<0.0001, *χ*^2^-test). Progression-free survival was 6.9 months for all pts irrespective of age. Patients ⩽65 years showed a PFS of 6.5 months as compared with 7.0 months for pts ⩾65 years (*P*=0.12, log-rank test; see Figure 2).

A total of 124 pts (18.9%) experienced grade III/IV non-skin-related toxicity during study treatment with cetuximab. Of these, 64 pts (51.6%) were in the age group 18–65 years and 60 pts (48.4%) were older than 65 years. In all, 9% of the pts suffered grade III/IV gastrointestinal toxicities, followed by 4.7% haematological toxicities and 3% hepatic toxicities. Infusion-related reaction grades III/IV were reported for five pts (0.8%). Table 2 shows grade III/IV toxicities comparing both age groups in detail. Severe adverse events related to cetuximab occurred in 2% of the pts with no significant difference between both age groups (*P*=0.68, *χ*^2^-test). One cetuximab-related event was life-threatening (allergic reaction).

Overall, there were 336 grade III/IV non-skin-related toxicities documented, of these 84.5% were grade III and 15.5% grade IV. The median duration of these toxicities was 7 days. The older pts suffered from a significantly longer duration with 9 days (range: 0–104 days) compared with the younger pts with 5 days (range: 0–104 days) respectively, (*P*=0.0004, Wilcoxon test). A total of 66.7% of these toxicities could be resolved either with or without supportive treatment, whereas 20.2% persisted past the observational period. A total of 6.3% (*n*=21) events led to permanent damage (among those one renal failure) and 6.8% (*n*=23) of the toxicities led to death. There was no significant difference between both age groups in this aspect (*P*=0.054, *χ*^2^-test). One patient died owing to gastric bleeding with heamatemesis after paracentesis.

Of note, 793 skin reactions were documented. In all, 69.7% of the pts showed any skin toxicity grades I–IV, 9.8% with severity of grades III/IV. Skin rash was the most common skin effect with a prevalence of 64.2% (83.7% grades I/II). There was no difference between the age groups in this aspect (*P*=0.34, *χ*^2^-test), however, the pts >65 years showed a trend towards higher grades of toxicity (*P*=0.05, *χ*^2^-test). A prophylactic skin treatment was initiated in only 12.5% of the pts by their treating physicians, with no difference in treatment between the age groups (*P*=0.58, *χ*^2^-test). Supportive therapy of skin reactions led to an improvement of symptoms in 83.2% of pts with topical and/or systemic therapy.

## Discussion

During the last decade, incremental improvement in the survival of pts with mCRC has been achieved, primarily through the addition of novel active therapeutic agents. The monoclonal antibody cetuximab is such a novel agent that has shown marked effectiveness in pretreated pts as well as in first-line pts when combined with chemotherapy (Vincenzi *et al*, 2006; Saltz *et al*, 2007). Elderly pts are usually not included in studies applying new treatment strategies. In fact, elderly pts are commonly significantly underrepresented in most phase II and III clinical trials, making meaningful conclusions about safety and efficacy difficult (Lewis *et al*, 2003). If elderly pts are included, they are generally selected for good performance status and minimal co-morbidities. However, this does not represent the usual population at such advanced stage and pretreatment of mCRC in standard general practice. Colorectal cancer is primarily a disease of the elderly, with a median age at diagnosis of 71 years. Even though most studies employing early 5-FU-based regimes have shown similar efficacy and tolerability in elderly compared with younger pts, chemotherapy is used less frequently in elderly pts (Folprecht *et al*, 2004; Lemmens *et al*, 2005). This study included a relatively large population of elderly pts (*n*=305) with a median age of 71 years. Most pts (78%) showed a reduced performance status with an ECOG of only 1–2 and significant comorbidities, as one would expect in an unselected population with advanced mCRC. This stands in contrast to the Bond and MABEL trials, which recruited pts in a similar therapeutic situation, but with better performance status: in the Bond trial, 87.7% of all pts showed a Karnofsky Performance Status (KPS) score of 80–100% (Cunningham *et al*, 2004). In the MABEL study, 69.2% of all pts showed a KPS of ⩾90% (Wilke *et al*, 2008).

Most pts had received at least one previous chemotherapy for mCRC. Cetuximab was mainly combined with irinotecan. Dosing schedules for irinotecan varied according to the approval status at that time, however, most pts received irinotecan on a weekly basis at a dose of 80 mg m^−2^. In all, 40% of the pts had received at least two previous chemotherapies for mCRC before cetuximab initiation. Interestingly, the RRs to the cetuximab-based therapy were similar between the two age groups. Previous studies report RRs of 20–26% and a PFS of about 4 months for combinations of cetuximab and chemotherapy in pretreated mCRC pts (Cunningham *et al*, 2004; Saltz *et al*, 2004). This study compares favourably, with a RR of 37% and a PFS of 6.9 months, with these historical studies. The pts treated in this NIS were not the usual preselected study population with a good ECOG performance status, they rather reflect real-life pts with advanced mCRC, seen in a wider general practice. However, comparing efficacy data with historical controls is always burdened by limitations. In addition to this being an uncontrolled observational study, the data of this NIS were not centrally or board-reviewed in contrast to the trials mentioned above. The follow-up was only 12 months, which may have left very rare events of late remissions and late toxicities undocumented. The most important limitation of this study, however, is the missing k-RAS evaluation. While the k-RAS status is tested in all on-going NIS, the importance of k-RAS and other parameters as predictive factors was not established at the time of trail commencement and for most of the initial study period, and hence was not be incorporated (Bokemeyer *et al*, 2009; Van *et al*, 2009, 2011; Ciardiello *et al*, 2011). In this regard, it should be reminded that both MABEL and Bond trials were also conducted without previous k-RAS testing.

In all, 64% of the pts developed a skin rash in response to cetuximab treatment. While the rash was inconvenient for most pts from an aesthetic point of view, it was rarely severe or resulted in termination of the treatment. There was no significant difference in prevalence of skin rash between both age groups, however, the elderly pts showed a trend towards higher grades and duration of toxicity. Consistent with other reports, the presence and severity of the skin rash was strongly related to the response to treatment (Perez-Soler and Saltz, 2005). In this respect, there were no differences between the younger and elderly treatment group either. Besides skin toxicity, 18.9% of the pts experienced other grade III/IV toxicities during study treatment. All toxicities of the cetuximab-based chemotherapy lay within acceptable and expected margins for treatment of advanced metastatic cancer. Importantly, there were no significant differences in those treatment-related toxicities between the younger and the elderly study group.

With the limitations noted, we believe that the data from this NIS can be seen as a helpful source of information for everyday clinical practice. Like other NIS, that is, the role of bevacizumab beyond progression in mCRC (Grothey *et al*, 2008), the results of this study on cetuximab underlines the importance to expand the age limit of randomised clinical phase III trials for mCRC pts to older ages, for example, <75 years, necessary to evaluate efficacy and safety in this age group.

### Conclusions

Increased survival time of pts with mCRC has led to older pts with reduced performance status eligible for second-, third- and further-line treatment options. This NIS reports one of the largest mCRC collectives >65 years of age and reduced performance status. Cetuximab has a similar efficacy and safety profile for pts aged ⩽65 and >65 years of age. The reduced performance status did not prevent oncologists from treating pts with cetuximab-containing chemotherapy schedules. Elderly pts with reduced performance status and comorbidities can be treated effectively and safely with cetuximab.

## Figures and Tables

**Table 1:** Baseline characteristics of pts based on age groups 18–65 years or >65 years

**Demographic data**	**Age 18–65 years (*n*=309)**	**Age >65 years (*n*=305)**	***χ*^2^-test**
*Age at study entry*			*P*=0.001
Median	59 years	71 years	
Range	23–65 years	66–89 years	
			
*Age at primary diagnosis*			*P*=0.009
Median	56 years	68 years	
Range	18–65 years	53–85 years	
			
*Gender (% of pts)*			*P*=0.65
Male	66	64	
Female	34	36	
			
*ECOG (% of pts)*			*P*=0.56
0	19	17	
1	61	59	
2	16	19	
3	4	4	
			
*Location of primary tumour (% of pts)*			*P*=0.81
Colon	59	60	
Rectum	41	40	
			
*Pre-existing medical disorder (% of pts)*			
Cardiovascular disease	19	31	*P*=0.0001
Diabetes	10	12	*P*=0.3
Allergies/hypersensitivity	7	5	*P*=0.6
Pulmonary disease	7	8	*P*=0.2
			
*CCI, %*			*P*=0.001
No comorbidity (score 0)	49 (*n*=151)	12 (*n*=36)	
Moderate comorbidity (score 1–2)	28 (*n*=87)	40 (*n*=122)	
Severe comorbidity (score ⩾3)	23 (*n*=71)	48 (*n*=146)	

Abbreviations: CCI=Charlson Comorbidity Index; pts=patients.

**Table 2:** Previous types of therapy and number of previous chemotherapy lines

**Previous therapy before cetuximab-based treatment**	**Age 18–65 years (*n*=309)**	**Age >65 years (*n*=305)**
Resection of primary tumour (% of pts)	94	95
Resection of metastasis (% of pts)	34	26
Radiation (% of pts)	30	19
Adjuvant chemotherapy (% of pts)	46	44
Chemotherapy for metastatic disease (% of pts)	95	96
		
*Number of previous chemotherapy lines (% of pts)*		
0	5	4
1	27	18
2	39	39
3	17	25
4	13	14
		
**Best response (% of pts)**	**Age 18–65 years (*n*=309)**	**Age >65 years (*n*=305)**
CR	3	3
PR	35	33
SD	31	34
PD	25	23
NA	7	7
		
**Grade III/IV toxicities (NCI criteria) in % of pts**	**Age 18–65 years (*n*=309)**	**Age >65 years (*n*=305)**
Heamatological toxicity	6	4
Febrile neutropenia	1	1
Allergic reaction	0.3	1
Gastrointestinal toxicity	10	9
Hepatic toxicity	3	4
Skin toxicity	8.8	10.9

Abbreviatons: CR=complete remission; NA=not available; NCI=National Cancer Institute; PD=progressive disease; PR=partial remission; pts=patients; SD=stable disease.

Best response according to age groups (CR, PR, SD, PD and NA) as well as best response according to previous chemotherapy line for both age groups. NCI toxicities according to age groups (grades III/IV).
